# Differences in Breast Cancer Presentation at Time of Diagnosis for Black and White Women in High Resource Settings

**DOI:** 10.1007/s10903-021-01161-3

**Published:** 2021-03-08

**Authors:** Jo-Ann Osei-Twum, Sahra Gedleh, Aisha Lofters, Onye Nnorom

**Affiliations:** 1 Department of Physical Therapy, University of Toronto, 500 University Avenue, Toronto, ON M5G 1V7 Canada; 2 Department of Family and Community Medicine, University of Toronto, 500 University Avenue, Toronto, ON M5G 1V7 Canada; 3 Women’s College Research Institute, 76 Grenville St, Toronto, ON M5G 1N8 Canada; 4 Dalla Lana School of Public Health, University of Toronto, 155 College St, Toronto, ON M5T 3M7 Canada

**Keywords:** Breast cancer, Race, Triple negative breast cancer, Canada, United States, United Kingdom

## Abstract

This paper provides a narrative review of the existing literature on differences in demographic and biological features of breast cancer at time of diagnosis between Black and White women in Canada, the United Kingdom and the United States. Electronic database searches for published peer-reviewed articles on this topic were conducted, and 78 articles were included in the final narrative review. Differences between Black and White women were compared for eight categories including age, tumour stage, size, grade, lymph node involvement, and hormone status. Black women were significantly more likely to present with less favourable tumour features at the time of diagnosis than White women. Significant differences were reported in age at diagnosis, tumour stage, size, grade and hormone status, particularly triple negative breast cancer. Limitations on the generalizability of the review findings are discussed, as well as the implications of these findings on future research, especially within the Canadian context.

## Introduction

Breast cancer continues to be a significant cause of morbidity and mortality for Canadian women. Breast cancer was responsible for a quarter of new cancer diagnoses in women and 13% of all cancer-related deaths in women in 2017 [1]. The influence of various social and demographic factors on the morbidity and mortality associated with breast cancer has been well established in the literature [2]. Race is one such factor, which in some contexts is used as a variable in analysis and reporting.

Race is a social construct rather than a biological determinant of health. However, the product of the construct of race—racism, experienced at an institutional and interpersonal level—has a profound and measurable impact on racialized individuals in all sectors of society including the health care system. In this review the term race is used as a proxy for racism and to denote two groups of women, those identified as *Black* and those defined as *White*, whilst recognizing the diversity of experiences within these categories. The influence of race and arguably racism on the experience of breast cancer amongst women in the United Kingdom (UK) and the United States (US) is particularly striking and well-established in the literature. Less is known about how breast cancer outcomes differ between racialized women in Canada, given the current lack of race-based data collected in the Canadian health care system. Significant differences in breast cancer incidence, diagnosis and prognosis have been demonstrated between ethnic and racial groups in the US and the UK [2]. Despite a greater incidence of breast cancer amongst White women [3], prognosis for Black women with breast cancer has been noted to be poorer in numerous studies [4–6]. Black women had a lower rate of overall 5-year survival in some studies [7], as well as lower rates of distant relapse-free survival [8]. Disease recurrence was also noted to be greater amongst Black women in the US diagnosed with earlier-stage breast cancers [9]. However, similar findings were not observed in other studies based in the US or the UK. For example, Roseland et al. [10] noted no differences in mortality for patients diagnosed with stages I–III breast cancer in Michigan when data was stratified by race. In another retrospective single centre study in London, UK by Bowen et al. [11], no significant difference in overall survival was noted by race.

A number of social and demographic factors have been proposed to contribute to these findings, including differences in age at diagnosis [12–14], geographic location [14], socioeconomic status [4, 12], as well as individual and regional differences in breast cancer screening [15]. In addition to these factors, a number of studies have demonstrated differences in tumour biology and have suggested that these differences may also contribute to differences in breast cancer prognosis between Black and White women. In particular, later cancer stage at time of diagnosis [5], larger tumour size [6], and higher incidence of triple negative breast cancers [8, 16] amongst Black women have been noted in a number of studies in both the UK and the US.

This paper sets out to conduct a narrative review of the existing literature regarding the differences in certain demographic and biological features of breast cancer at the time of diagnosis, including age, tumour size, grade, hormone receptor status, and lymph node involvement, between Black and White women in the UK, Canada and the US. These features in particular are known to be associated with breast cancer prognosis, with poorer prognosis for patients with larger tumours at the time of diagnosis, triple negative hormone status, and axillary lymph node involvement [17–19].

Investigating the association of race and racism on these features of malignant breast tumours is particularly challenging in the Canadian context, where there is a lack of surveillance data that examines health outcome disparities among racialized groups, particularly amongst Black women. Surveillance data that includes information about race and ethnicity is collected in the US through organizations like the Surveillance, Epidemiology, and End Results (SEER) Program and the National Cancer Database (NCDB). In the UK, with a centralized publicly funded healthcare system that is more similar to the Canadian model of healthcare, race data is similarly collected through the National Health Service (NHS). Reviewing the existing UK and US literature can provide general trends in the characteristics of breast tumours in women belonging to these racialized groups, and may shed some light on the etiology of the aforementioned differences in diagnosis and prognosis. The implications of the findings of this narrative review are key for improved equity in breast cancer prevention, screening, and diagnosis and can guide future endeavours in research regarding breast cancer in the Canadian context.

## Methods

A research librarian conducted electronic database searches of Ovid MEDLINE, Ovid EMBASE, Ovid EMB Reviews, CINAHL, and Web of Science. Searches were limited to English language reports and peer-reviewed literature from Canada, the US and the UK, published between 2005 and 2016. Search terms and keywords used with these bibliographic databases included but were not limited to: “African American”, “Black”, “Caribbean”, “non-White”, “breast neoplasms”, “carcinoma, lobular”, “tumour”, “age factors”, “delayed diagnosis”, “neoplasm grading”, “neoplasm invasiveness”, and “health status disparities”. For the Ovid MEDLINE search, similar terms were combined using the Boolean operator OR and separate concepts were combined using the Boolean operator AND. This initial search yielded a total of 6,434 results, which were reduced to 3215 entries following the removal of duplicate publications using EndNote.

**Fig. 1:**
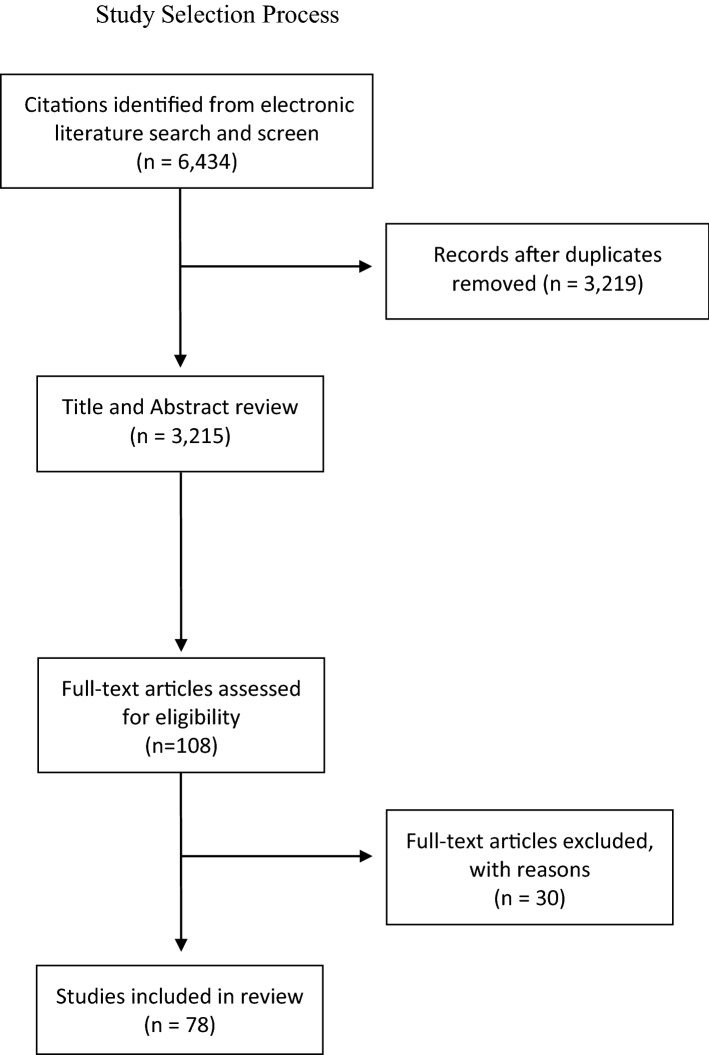
Study selection process

Two authors (SG, JO) reviewed titles and abstracts of these publications to identify those that met inclusion criteria for full-text review (see Fig. 1). Eligibility was determined using the research question and was limited to articles reporting breast cancer characteristics at the time of diagnosis for Black women compared to White women. Articles that included other racialized groups in addition to Black and White women were also included. This preliminary screen identified 108 publications for full-text review. Details of the study design, population, time period, variables, outcomes, age at diagnosis, tumour type, stage of tumour, and prognosis were abstracted to a Microsoft Excel spreadsheet by the authors. Of these, one article could not be accessed through the University of Toronto library system and a further 29 publications were deemed irrelevant, upon review. As such, the final number of publications included in this narrative literature review was 78 [2–14, 20–81].

## Results

### Description of Studies

Almost all of the studies included in this study were conducted in the US, with two of the included studies conducted in the UK, and no studies from Canada met the inclusion criteria (see Table 1). There was a great deal of geographical variation within the US, with study locations including New York, California, Florida, Georgia, Utah, and Illinois. The studies included were published between 2005 and 2016, including patients diagnosed with breast cancer between 1975 and 2014.

**Table 1:** Summary of age and stage at diagnosis for black and white women

References	Location	Sample size	Study design	Study population	Results		How race data was obtained
					Age at diagnosis	Stage	
*Diagnosis at younger age (Black women)*							
Anderson et al. [43]	United States	n = 440,653 Black: 34,478 White: 381,122	Retrospective	Women diagnosed with invasive breast cancer	Black women – 57.6 years vs. White women – 62.6 years (p < 0.001)	–	From SEER database
Barcenas et al. [5]	United States	n = 1178 Black: 489 White: 670 Asian/Pacific Islander: 12 Other/unknown: 7	Retrospective	Women diagnosed with breast cancer	Black women significantly more likely to be diagnosed at a younger age vs. white women (p = 0.00278)	Black women were diagnosed at later stages than white women, stage III (17.4% vs 12.2%) and stage IV (11.2% vs 9.1%) (p = 0.00454)	Not reported
Bharat et al. [35]	United States	n = 3596 Black: 496 White: 2917 Other/unknown: 183	Prospective database, with retrospective analysis	Women treated for invasive breast cancer or DCIS	Women diagnosed at a yzounger age ≤ 40 years were more likely to be Black (OR 2.25, 95% CI 2.17–2.53)	–	Not reported
Bowen et al. [11]	United Kingdom	Black: 102 White: 191	Retrospective	Women diagnosed with invasive breast cancer, age ≥ 16 years	Black women – 46 years White women – 67 (p = 0.001)	–	Self-report
Chen and Li [68]	United States	Black: 10,874 Non-hispanic white: 72,623 hispanic white: 9944 Asian/Pacific islander: 8068 American Indian/Alaska Native: 555	Retrospective	Women aged ≥ 20 years	20–49 years: Black women – 26.9% vs. White women – 17.6% 50–64 years: Black women – 40.4% vs. 37.3%	–	From SEER database
Cunningham et al. [20]	United State, South Carolina and Ohio	South Carolina Black: 5498 White: 18,420 Ohio Black: 6528 White: 64,713	Retrospective	Women of European or African descent aged greater than 15 years diagnosed with invasive breast cancer	After age adjustment black women diagnosed 1–2 years earlier on average than white women	–	From medical records
DeSantis et al. [4]	United States	n = 193,969 Black: 24,483 White: 169,486	Retrospective	Black and white women (aged between 20 and 99 years)	Black women were more likely to be diagnosed at a younger age	Greater % of stage II tumours for black women (32.9%) vs white women (27.9%), greater % of stage III tumours for black women (14.6%) vs white women (9.5%), greater % of stage IV tumours for black women (4.6%) vs white women (2.5%)	From hospital records
Gnerlich et al. [32]	United States	n = 243,012 Black: 20,389 White: 204,416 Other/Unknown: 18,207	Retrospective	Women diagnosed with primary breast cancer	Black women diagnosed < 40 years – 14.1% vs. > 40 years – 8% White women diagnosed < 40 years – 75.6% vs. > 40 years – 84.7% (p < 0.001)	–	From SEER database
Iqbal et al. [66]	United States	Black: 38,751 Non-hispanic white: 268,675 Hispanic white: 34,928 Chinese: 4937 Japanese: 3751 South Asian: 2191 Other Asian: 14,332 Other: 5998	Retrospective	Women diagnosed with first invasive breast cancer	Median age: Black women – 57 years vs. white women – 61 years	Stage I: Black women – 37.0% vs. White women – 50.8% (OR 0.56, 95% CI 0.64–0.67, p < 0.001)	From SEER database
Kwan et al. [13]	United States	Black: 155 White: 1943	Prospective	Women diagnosed with diagnosed with early stage invasive breast cancer, aged 18–70 years	Black women diagnosed < 50 years – 27.7% White women diagnosed < 50 years – 20% (p < 0.0001)	-	Self-report
Lund et al. [80]	United States	Black: 814 White: 967	Retrospective	Women diagnosed with primary invasive breast cancer	≤ 50 years: Black women – 31.7% vs. White women – 21.7% Mean age: Black women – 56.9 years vs. White women – 61.2 years	Stage III: Black women – 11.8% vs. white women – 6.8% Stage IV: Black women – 7.7% vs. white women – 2.7% (p < 0.001)	From Atlanta SEER registry and Georgia Comprehensive Cancer Registry
McBride et al. [7]	United States	n = 256,174 Black: 21,861 White: 234,313	Retrospective	Women diagnosed with stage I–IIIa invasive breast cancer	Black women – 55 years White women – 60 years (no statistical analysis)	T1: Black women – 52.2% vs. white women – 65.9% T2: Black women – 40.4% vs. white women – 30.1% T3: Black women – 7.4% vs. white women – 4.0% (p < 0.0001)	From SEER database
Monzavi-Karbassi et al. [64]	United States, Arkansas	Black: 208 White: 869	Retrospective	Black and white women receiving breast cancer treatment	< 50 years: Black women – 46.2% vs. white women – 30.6% (p < 0.001)	Stage II: Black women – 43.8% vs. White women – 41.1% (p < 0.001) Stage III: Black women – 18.8% vs. White women – 12.8% (p < 0.001) Stage IV: Black women – 10.6% vs. White women – 4.7% (p < 0.001)	From Arkansas tumor registry files
Moran et al. [9]	United States	Black: 207 White: 2164	Retrospective	Women diagnosed with early stage breast cancer	≤ 40 years: Black women – 20% White women – 12% (p = 0.016)	–	Self-report
Robbins et al. [87]	United States	Black: 5815 White: 38,301	Retrospective	Women diagnosed with invasive cancers, aged ≤ 84 years	Mean age: Black women – 60.3 years vs. White women – 61.1 years (p < 0.001)	–	From SEER database
Roberts et al. [63]	United States, North Carolina	Black: 609 White: 859	Retrospective	Women diagnosed with ER^+^, stage I or II and HER2^−^ breast cancer	Black women – 55.5 years vs. white women – 57.7 years (p = 0.003)	–	Self-report
Sachdev et al. [78]	United States, Tennessee	Black: 88 White: 36	Retrospective	Women diagnosed and receiving treatment for triple negative invasive breast cancer	Median age: Black women – 49.5 years vs. white women – 55 years (p = 0.024)	No significant difference in stage at diagnosis (p = 0.21)	From medical records
Sassi et al. [49]	United States	Black: 23,689 White: 311,842	Retrospective	Women diagnosed with breast cancer	Black women – 58.6 years vs. white women – 63.3 years (no statistical analysis)	–	Not reported
Schootman et al. [27]	United States	Black: 2101 White: 32,387 Other: 1320	Retrospective	Women > 66 years diagnosed with distant metastases from primary breast cancer	Black women more likely to be diagnosed with distant metastases from primary breast cancer at younger ages than White women	–	From SEER database
Short et al. [77]	United States	Black: 99 White: 476	Retrospective	Women newly diagnosed with breast cancer	Mean age: Black women – 48.9 years vs. White women − 52.9 years (p = 0.001)	Stage IV: Black women – 6.1% vs. white women – 3.2% (p < 0.05) After adjustment, diagnosis with later stage cancer OR 1.71, 95% CI 1.09–2.76, (p = 0.02)	From medical charts
Swede et al. [62]	United States, Connecticut	n = 416 Black: 202 White: 214	Retrospective	Women diagnosed and receiving treatment for breast cancer	Black women – 54.8 years vs. 58.4 years (p = 0.007)	SEER stage, distant: Black women – 5.0% vs. White women – 1.4% (p = 0.04)	From patient chart
Tao et al. [6]	United States, California	Black: 9738 White: 93,760	Retrospective	Women diagnosed with invasive breast cancer	Mean age: Black women – 58.8 years vs. white women – 62.3 years (p < 0.05)	Stage III: Black women – 15.4% vs. white women – 11.0% Stage IV: Black women 7.5% vs. white women – 4.6% (p < 0.05)	From medical record
Thomas et al. [60]	United States	Non-hispanic black: 33,301 Non-hispanic white: 241,236 Non-hispanic Asian/Pacific Islander: 9508 Hispanic: 15,782	Retrospective	Women diagnosed with invasive breast cancer	< 30 year: Black women – 1.3% vs. white women – 0.5% (p < 0.001)	Stage I: Black women – 58.6% vs. white women – 68.2% (p < 0.001)	From National Cancer Database
Vicini et al. [75]	United States, Michigan	n = 699 Black: 39 White: 660	Retrospective	Women diagnosed with invasive breast cancer	≤ 50 years: Black women – 49% vs. white women – 26% (p = 0.002)	Stage IIB: Black women – 31% vs. White women – 10% (p < 0.001)	Self-report
Woods et al. [2]	United States	n = 5751 Black: 632 White: 5119	Retrospective	Women diagnosed with breast cancer	Black women – 56.4 years vs. white women – 58.6 years (p < 0.01)	Black women less likely to be diagnosed with Stage I tumour (OR 0.80, 95% CI 0.67–0.96, p = 0.02) and more likely to be diagnosed with Stage 3 tumour (OR 1.50, 95% CI 1.11–2.01, p = 0.01) vs. white women	From patient, patient chart or treating physician
Yang et al. [23]	United States, Florida	n = 935 Black: 130 White: 777 Asian/Pacific Islander/Native American: 13 Not reported: 15	Retrospective	Women diagnosed with inflammatory breast cancer	Black women were diagnosed < 45 years (28.5%) vs. white women (18.3%) (p = 0.003)	No significant difference in tumour stage at diagnosis between black and white women (p = 0.260)	From cancer registry and hospital records
*Incidence rate ratio*							
Baquet et al. [42]	United States	n = 171,372 Black: 15,877 White: 155,495	Retrospective	Women diagnosed with breast cancer	Significantly higher incidence among Black women < 40 years, incidence rate ratio – 1.16, 95% CI 1.10–1.23	Advanced stage: Black women – 9.0% vs. white women – 5.3% (p < 0.0001) Regional stage: Black women – 34.2% vs. 27.8% (p < 0.0001)	From SEER database
Joslyn et al. [55]	United States	n = 363,801 Sample size for Black and White women not provided	Retrospective	Women diagnosed with invasive breast cancer, aged ≥ 10 years	*Cross-over effect*: significantly higher incidence rate of breast cancer for black women < 40 years (20–39 years, 95% CI > 1.0).and significantly lower incidence rate > 50 years (50 + years, 95% CI < 1.0) compared to white women	*Local-*significantly lower incidence rate of localized tumours amongst black women > 40 years (95% CI < 1.0) compared to white women *Distant*-significantly greater incidence rate of distant stage tumours amongst black women at all ages (95% CI > 1.0) compared to white women	From North American Association of Central Cancer Registries Breast Cancer Research Dataset
*No difference in age at diagnosis*							
Aggarwal et al. [69]	United States, Indiana	Black: 325 White: 675	Retrospective	Women diagnosed with breast cancer, ≥ 65 years	Mean age: Black women – 74.5 years vs. white women – 74.0 years (p = 0.29)	Stage I: Black women – 15.4% vs. White women – 29.5% (p < 0.001) No significant difference for other stages	Self-report
Chu et al. [22]	United States	Black: 252 White: 123	Prospective	Low income Black and White women with Stage 0-III, ER- breast cancer, receiving standardized treatment	Mean age of diagnosis was not significantly different between black women, 55 years vs white women 59 years (p = 0.25)	No significant difference in stage at diagnosis between black and white women (p = 0.29)	Not reported
Copson et al. [8]	United Kingdom	n = 2915 Black: 118 White: 2690 Asian: 87	Prospective	Women diagnosed and receiving treatment for breast cancer, aged ≤ 41 years	Median age: Black women – 36 years vs. white women – 36 years (p = 0.463)	–	Self-report
Crowe et al. [57]	United States	Black: 313 White: 2012	Prospective	Women diagnosed with invasive breast cancer with available 2000 census tract data	Median age – 57 years, no significant difference (p = 0.37)	Stage I: Black women – 43% vs. white women – 54% Stage II: Black women – 44% vs. white women – 38% Stage III: Black women – 8% vs. white women – 6% Stage IV: Black women – 5% vs. white women – 3% (p = 0.002)	Self-report
George et al. [67]	United States	Black: 304 White: 330	Retrospective	Black and white women ≤ 85 years	No significant difference in age at diagnosis < 55 years: Black women – 46.4% vs. white women – 52.1%, p = 0.1487	–	From medical records
Jiagge et al. [82]	United States, Ghana and Ethiopia	Black: 272 White: 321 Ghanaian: 234 Ethiopian: 94	Retrospective	Women diagnosed with invasive breast cancer	Significantly lower for Ghanaian women – 49 years and Ethiopian women – 43 years vs. African American women – 60 years and White women – 62 years (p < 0.001)	No significant difference in stage at diagnosis (p = 0.4986)	Not reported
Lund et al. [39]	United States	Black: 176 Non-Black: 23	Retrospective	Women diagnosed with invasive breast cancer	Black women – 58 years Non-Black women – 57 years (p = 0.967)	–	Self-report
Maloney et al. [50]	United States	n = 52 Black: 36 White: 16	Retrospective	Women diagnosed with breast cancer, uninsured and below poverty line	No significant difference between age at diagnosis for Black women – 56.1 years and white women – 56.2 years (p = 0.98)	–	Not reported
Marti et al. [38]	United States	n = 215 Black: 29 White: 31 Asian: 53 Hispanic: 102	Prospective database, retrospective analysis	Women diagnosed with invasive breast cancer or DCIS, of low socioeconomic status	Invasive breast cancer: Black women – 56 years White women – 53 years (p = 0.009) DCIS: Black women – 51 years White women – 63 years (p = 0.08)	No significant difference is stage at diagnosis for both invasive breast cancer (p = 0.74) and DCIS (p = 0.80)	Not reported
Rizzo et al. [28]	United States	Black: 93 Non-Black: 14	Retrospective	Women diagnosed with stage III breast cancer	Black women – 55 years Non-Black women – 53.1 years (p = 0.63)	No significant difference in stage at diagnosis (p = 0.39)	From medical records
Roseland et al. [10]	United States	Black: 818 White: 1569	Retrospective	Women diagnosed with Stage I, II or III breast cancer	No significant difference in age at diagnosis (p = 0.3314)		Not reported
Stark et al. [37]	United States	n = 1263 Black: 441 White: 822	Retrospective	Women diagnosed with primary invasive breast cancer	Black women – 60.3 years White women – 62.4 years (p = 0.051)	Stage IV: Black women – 7.8% White women – 3.1% (p = 0.002)	Self-report
*Diagnosis at older age (Black women)*							
Chagpar et al. [74]	United States, Kentucky	n = 1903 Black: 469 White: 1,145	Retrospective	Women diagnosed with hormone receptor positive breast cancer	Median age: Black women – 57 years vs. White – 55 years (p = 0.032)	–	Not reported
Nassar et al. [30]	United States	Black: 217 White: 141	Retrospective	Women diagnosed with primary ductual carcinoma in situ with focal invasion > 1 mm	Black women – 60 years White women – 56 years (p = 0.001)	–	From medical records and SEER database
*Stage only*							
Chlebowski et al. [58]	United States	n = 156,570 Diagnosed with breast cancer: 3938 Black: 242 White: 3455 Other: 202 Unknown: 39	Prospective	Post-menopausal women aged 50–79 years	–	No significant difference in tumour stage at diagnosis (p = 0.39)	Self-report
Hahn et al. [47]	United States, Georgia	n = 829 Black: 250 White: 579	Retrospective	Women diagnosed with unilateral invasive breast cancer	–	No significant difference in stage at diagnosis between Black and White women after adjusting for all variables (p = 0.29)	Self-report
Warner et al. [71]	United States	Black: 1718 White: 17,696 Hispanic: 1363 Asian: 650	Retrospective	Women diagnosed with Stage I, II, III or IV breast cancer	–	Stage III or IV: Black women – 26% vs. White women – 15% (p < 0.0001) (OR 1.50, 95% CI 1.29–1.74)	Self-report

Most of the included studies were retrospective and observational studies. There were eight prospective studies included, with an additional two studies utilizing prospective databases. The sample sizes varied widely in the included studies and ranged from a few hundred participants to hundreds of thousands of participants. Thirty-three of the included studies utilized data collected nationally by the SEER Program and the NCDB in the US. The remaining 45 studies utilized data from single centres, or multiple centres within a specific geographical region.

One of the studies included from the UK was a retrospective observational study conducted using data from a single East London hospital, enrolling a total of 445 participants [11]. The second UK study was a prospective cohort study, obtaining data from the medical records of 2956 patients at 127 hospitals across the UK [8].

### Age at Diagnosis

Forty-four of the 78 articles included in this narrative review analyzed differences in age at diagnosis stratified by race (Table 1). Race was significantly associated with age at diagnosis in the thirty-two of these papers, and Black women were generally found to be diagnosed with breast cancer at a younger age than White women.

Method of reporting age varied substantially, with 28 of the articles reviewed using the median or mean age at diagnosis to analyze differences by race. Other methods of analysis include comparison of the incidence rate of breast cancer by age group [42, 55] and analysis of the proportion of breast cancer by race within given age brackets. The range of mean age at diagnosis for Black and White women were similar, ranging from 36 years [8] to 74.5 years [69]. However, Black women were younger than their White counterparts at diagnosis in thirty of the studies reviewed. A similar trend was noted in the incidence of breast cancer in younger age brackets. Black women were more likely to be diagnosed before the age of 50 [9, 32, 60, 75], and a higher incidence of breast cancer was noted amongst Black women before the age of 40 [42, 55].

Twelve of the articles reviewed found no statistically significant difference in average age at diagnosis by race. Most of these papers reported an average age at diagnosis between 50 and 65 years of age for both Black and White women [22, 28, 37–39, 50, 57, 82]. Additionally, two studies found that Black women were more likely to be diagnosed at an older age than White women [30, 74]. Of significance, Nassar et al. [30] looked at the age of diagnosis for ductal carcinoma in situ (DCIS), finding that Black women were diagnosed at a significantly older age (60 years) compared to White women (56 years; p < 0.001). However, another article in this review also looked at incidence of DCIS by race and noted that Black women were more likely to be diagnosed with DCIS at a younger age (≤ 40 years old) than White women [35].

### Stage at Diagnosis

Breast cancer staging describes the degree of metastasis and disease progression. The reviewed literature reported breast cancer stage at the time of diagnosis either using a scale from 0 to IV or by describing tumour stage as local, regional or distant. Sixteen publications reported a significant difference in breast cancer stage between Black and White women, whilst no significant difference was observed in nine studies (Table 1).

Black women were significantly less likely to be diagnosed at earlier stages (I and II) of breast cancer compared to White women [2, 14, 66, 69]. An additional six studies found that a greater proportion of Black women were diagnosed with stage II, III, or IV breast cancers compared to White women [6, 7, 64, 71, 75, 80]. Warner et al. [71] found that 20% and 6% of Black women were diagnosed with stage III (n = 344/1718) and IV (n = 103/1718) breast cancers, compared to 11% and 4% of White women (n = 1947/17,696; n = 708/17,696) (p < 0.0001). Furthermore, the odds of Black women being diagnosed with stage III or IV tumours was significantly greater than White women (OR 1.34, 95% CI 1.16–1.56). Similarly, Stark et al. [37] and Short et al. [77] found that a greater proportion (4.7% and 2.5%) of Black women were diagnosed with stage IV breast cancer compared to White women.

Two studies described breast cancer stage based on localization of the tumour and in both instances, differences were observed between Black and White women. Black women were significantly more likely to present with distant breast cancers compared to White women [5, 62]. However, it is important to note that nine studies reported no significant difference in stage at diagnosis between Black and White women [12, 22, 28, 39, 47, 58, 70, 78, 82].

### Tumour Size

Twenty-seven articles included in this review analyzed tumour size at diagnosis. Of these articles, twenty articles indicated a significant difference in tumour size by race (Table 2). There was heterogeneity in the method of reporting tumour size. Fourteen articles used ranges of measurements similar to those found in the TNM classification system, the most commonly used system for tumour classification and gold standard of measurement. For this classification system, size was reported as ≤ 2 cm (T1), 2–5 cm (T2), or > 5 cm (T3 or greater). Of the remaining studies, ten compared results by mean size (cm).

**Table 2:** Summary of tumour size and grade at diagnosis for black and white women

References	Location	Sample size	Study design	Study population	Results		How race data was obtained
					Tumour size	Tumour grade	
Ambrosone et al. [36]	United States	Cases: 1119 Black: 559 White: 560 Control: 858 Black: 412 White: 446	Multi-center case–control	Women diagnosed with invasive breast cancer or primary DCIS, aged 20–75 years	–	Poorly differentiated tumours: Black women – 51.6% White women – 32% (p < 0.05)	Self-report
Anderson et al. [43]	United States	n = 440,653 Black: 34,478 White: 381,122	Retrospective	Women diagnosed with invasive breast cancer	Black women – 2.8 cm vs. white women – 2.1 cm (p < 0.001)	Significantly higher incidence of high grade tumours for black women, IRR = 1.1 (95% CI 1.1–1.2)	From SEER database
Baquet et al. [42]	United States	n = 171,372 Black: 15,877 White: 155,495	Retrospective	Women diagnosed with breast cancer	–	Poorly differentiated: Black women – 43.6% White women—29.7% (no p value)	From SEER database
Bowen et al. [11]	United Kingdom	n = 293 Black: 102 White: 191	Retrospective	Women diagnosed with invasive breast cancer, age ≥ 16 years	No significant difference in tumour size at diagnosis (p = 0.2)	Grade 1: Black women – 6% White women – 12% (p = 0.02) Grade 3: Black women – 62% White women – 42% (p = 0.02)	Self-report
Chagpar et al. [74]	United States	n = 1,205 Black: 262 White: 927 Other: 16	Retrospective	Women diagnosed with hormone receptor positive breast cancer	Median diameter: Black women – 1.9 cm White women – 1.7 cm (p = 0.009)	No significant difference in tumour grade at diagnosis	From Kentucky Cancer Registry
Chen and Li [68]	United States	n = 102,064 Black: 10,874 White: 72,623 Other: 18,567	Retrospective	Women aged ≥ 20 years	≥ 5.0 cm: Black women – 13.4% White women – 8.2% (no p value)	-	From SEER database
Chlebowski et al. [58]	United States	n = 156,570 Diagnosed with breast cancer: 3,938 Black: 242 White: 3,455 Other: 202 Unknown: 39	Prospective	Post-menopausal women aged 50 – 79 years	No significant difference in tumour size at diagnosis (p = 0.12)	Poorly differentiated: Black women – 43% White women – 25% Well differentiated: Black women – 13% White women – 25% (p < 0.001)	Self-report
Chu et al. [22]	United States	n = 375 White: 123 Black: 252	Prospective	Low income Black and White women with Stage 0-III, ER negative breast cancer receiving treatment	No significant difference in mean tumour size at diagnosis (p = 0.19)	No significant difference in tumour grade at diagnosis (p = 0.32)	From database
Copson et al. [8]	United Kingdom	n = 2956 Black: 106 White: 2690	Prospective	Women diagnosed and receiving treatment for breast cancer, aged ≤ 41 years	Median diameter: Black women – 2.6 cm White women – 2.2 cm (p = 0.0103)	Grade 3: Black women – 68.1% White women – 60.4% (non sig)	Self-report
Crowe et al. [57]	United States	n = 2325 Black: 313 White: 201	Prospective	Women diagnosed with invasive breast cancer with available 2000 census tract data	No significant difference in tumour size at diagnosis (p = 0.08)	–	Self-report
Cunningham et al. [20]	United States	n = 95,159 Black: 12,026 White: 83,133	Retrospective	Women of European or African descent aged greater than 15 years diagnosed with invasive breast cancer	–	Grade 1: Black women – 10–14% White women – 21–22% p < 0.001 Grade 3: Black women – 52–58% White women – 37–39%	From medical records
DeSantis et al. [4]	United States	n = 193,969 Black: 24,483 White: 169,486	Retrospective	Black and white women (aged between 20 and 99 years)	Black women diagnosed with larger tumours (OR 1.87, 95% CI 1.80–1.95)	Black women diagnosed with less differentiated tumours (OR 2.55, 95% CI 2.44–2.66)	From hospital records
George et al. [67]	United States	n = 634 Black: 304 White: 334	Retrospective	Black and White women ≤ 85 years	> 2.0 cm: Black women – 39.8% White women – 22.7% (p < 0.0001)	Poorly differentiated: Black women – 42.4% White women – 28.2% (p = 0.0005)	From patient chart
Hahn et al. [47]	United States	n = 829 Black: 250 White: 579	Retrospective	Women diagnosed with unilateral invasive breast cancer	–	No significant difference in grade at diagnosis between Black and White women	Self-report
Hance et al. [56]	United States	n = 180,224 Black: 14,196 White: 155,820	Retrospective	Women diagnosed with breast cancer	–	Black women are at a lower risk of diagnosis with a lower grade cancer (T1–T3) compared to white women (RR:0.80, 0.79–0.82)	From SEER database
Iqbal et al. [66]	United States	n = 373,563 Black: 38,751 White: 268,675	Retrospective	Women diagnosed with first invasive breast cancer	–	Distant: Black women – 1.5% White women – 1.0% (p < 0.001)	From SEER database
Jiagge et al. [82]	United States	Black: 272 White: 321 Ghanaian patients: 234 Ethiopian patients: 94	Retrospective	Women diagnosed with invasive breast cancer	–	Grade I: African American – 12.3% White women – 24.9% Grade II: African American – 37.3% White women – 41.3% Grade III: African American – 50.4% White women – 33.7% (p < 0.0001)	From medical records
Katz et al. [81]	United States	n = 1341 Black: 430 White: 911	Retrospective	Women diagnosed with breast cancer	> 5.0 cm: Black women 19.1% White women – 8.7% (p < 0.0001)	–	Self-report
Kenney et al. [40]	United States	n = 184 Black: 70 White: 98 Other: 16	Retrospective	Women with invasive breast cancer	< 50 years: Black women – 3.1 cm ≥ 50 years: Black women – 2.3 cm (p < 0.05)	–	Self-report
Lund et al. [39]	United States	n = 190 Black: 167 White: 16 Other: 7	Retrospective	Women diagnosed with invasive breast cancer	–	No significant difference in grade at diagnosis (p = 0.099)	Self-report
Lund et al. [80]	United States	n = 1842 Black: 814 White: 967	Retrospective	Women diagnosed with primary invasive breast cancer	2.1–5.0 cm: Black women – 33.7% White women 22.9% ≥ 5.0 cm: Black women – 9.6% White women – 3.6% (p < 0.001)	–	From Atlanta SEER registry and Georgia Comprehensive Cancer Registry
Maloney et al. [50]	United States	n = 52 Black: 36 White: 16	Retrospective	Women diagnosed with breast cancer, uninsured and below poverty line	No significant difference in size of tumour at diagnosis (p = 0.91)	–	From database
Marti et al. [38]	United States	n = 215 Black: 29 White: 31 Other: 155	Prospective database, retrospective analysis	Women diagnosed with invasive breast cancer or DCIS, of low socioeconomic status	Black women – 3.0 cm White women – 2.59 cm (p = 0.04)	–	From medical records
McBride et al. [7]	United States	n = 256,174 Black: 21,861 White: 234,313	Retrospective	Women diagnosed with Stage I–IIIa invasive breast cancer	Black women – 2.5 cm White women – 1.6 cm (p < 0.0001)	Incidence of high grade tumours: Black women – 45.7% White women – 31.9% (p < 0.0001)	From SEER database
Monzavi-Karbassi et al. [64]	United States	n = 1077 Black: 208 White: 869	Retrospective	Black and white women receiving breast cancer treatment	–	Grade III: Black women 41.8% White women – 4.8% Grade IV: Black women – 35.0% White women – 2.1% (p = 0.02)	From Arkansas tumour registry
Moran et al. [9]	United States	n = 2371 Black: 207 White: 2,164	Retrospective	Women diagnosed with early stage breast cancer	T2 (2.1–5 cm): Black women – 32% White women – 18% (no p value)	–	Self-report
Morris et al. [44]	United States	n = 199,504 Black: 16,853 White: 162,768	Retrospective	Women diagnosed with breast cancer	–	Black women more likely to be diagnosed with high grade tumours (p < 0.001)	From SEER database and hospital records
Nassar et al. [30]	United States	n = 358 Black: 217 White: 141	Retrospective	Women diagnosed with primary ductual carcinoma in situ with focal invasion > 1 mm	Black women – 1.83 cm White women – 1.15 cm (p = 0.001)	No significant difference in tumour grade at diagnosis	From SEER database and hospital records
Rizzo et al. [28]	United States	n = 107 Black: 93 Non-black: 14	Retrospective	Women diagnosed with stage III breast cancer	–	High grade: Black – 44.3% Non-Black 14.2% (p = 0.04)	From SEER database and patient chart
Roberts et al. [63]	United States	n = 1468 Black: 609 Non-Black: 859	Retrospective	Women diagnosed with ER^+^, stage I or II and HER2^−^ breast cancer	T2 (2.1–5 cm): Black women 32.3% non-Black women – 22.4% (p < 0.001)	Grade III: Black women – 24.9% non-Black women – 15.6% (p < 0.001)	Self-report
Roseland et al. [10]	United States	n = 2387 Black: 818 White: 1569	Retrospective	Women diagnosed with Stage I, II or III breast cancer	2.1–5.0 cm: Black women – 32% White women – 28% > 5.0 cm: Black women 9% vs. White women – 4% (p < 0.001)	Poorly differentiated: Black women – 45% White women – 32% (p < 0.0001)	From several databases
Sachdev et al. [78]	United States	n = 124 Black: 88 White: 3	Retrospective	Women diagnosed and receiving treatment for triple negative invasive breast cancer	–	No significant difference in tumour grade at diagnosis (p = 0.99)	From medical records
Stark et al. [37]	United States	n = 1263 Black: 441 White: 822	Retrospective	Women diagnosed with primary invasive breast cancer	Black women – 2.34 cm White women – 1.93 cm (p < 0.001)	Grade I: Black women – 19.6% White women – 30.3% Grade III: Black women – 45.2% White women – 29.3% (p < 0.001)	Self-report
Stark et al. [76]	United States, Ghana	Black: 581 White: 1008 Ghanaian women: 75	Retrospective	Women diagnosed with breast cancer	–	Grade III: African American – 44.9% White – 29.3% (p = 0.007)	Self-report
Stead et al. [25]	United States	n = 415 White: 148 Black: 177 Hispanic: 43 Other: 47	Retrospective	Women diagnosed with invasive breast cancer	No significant difference in tumour size at diagnosis (p = 0.64)	–	Self-report
Swede et al. [62]	United States	n = 416 Black: 202 White: 214	Retrospective	Women diagnosed and receiving treatment for breast cancer	No significant difference in tumour size at diagnosis (p = 0.22)	Grade III/IV: Black women – 50.3% White women – 42.7% (p = 0.04)	From tumour registry
Tao et al. [6]	United States	n = 103,498 Black: 9,738 White: 93,760	Retrospective	Women diagnosed with invasive breast cancer	T2 (2.01–5 cm): Black women – 34.5% White women – 29.1% (p < 0.05)	–	From medical record
Thomas et al. [60]	United States	n = 299,827 Black: 33,301 White: 241,236 Other: 25,290	Retrospective	Women diagnosed with invasive breast cancer	Black women – 2.54 cm White women – 2.07 cm (p < 0.001)	Poorly differentiated: Black women – 47.9% White women – 29.8% (p < 0.001)	From Natioanl Cancer Database
Vicini et al. [75]	United States	n = 699 Black: 39 White: 660	Retrospective	Women diagnosed with invasive breast cancer	Black – 1.7 cm White – 1.4 cm (p = 0.032)	Grade III: Black women – 52% White women – 29% (p = 0.006)	Self-report
Yang et al. [12]	United States	n = 63,472 White: 57,435 Black: 4,804 Hispanic: 5553	Retrospective	Women diagnosed with invasive breast cancer	Black women had significantly larger tumours (p < 0.001)	Higher grade tumours: Black women – 58.1% White women – 36.8%, (p < 0.001)	From cancer registry and hospital records
Yang et al. [23]	United States	n = 935 Black: 130 White: 777 Other: 13 Unknown: 15	Retrospective	Women diagnosed with inflammatory breast cancer	No significant difference in tumour size at diagnosis (p = 0.214)	Higher grade tumours: Black women – 92.4% White women – 78.1% (p = 0.003)	From cancer registry and hospital records

Using different size cut-off points for comparison, Black women tended to be diagnosed with larger tumours compared to White women. Based on the TNM classification system, Black women were more likely to be diagnosed with larger (T3 or greater) tumours [22] and were significantly more likely to be diagnosed with tumours ≥ 5 cm compared to White women [4, 5, 81]. Using a lower measurement of ≥ 2 cm, a large proportion of women diagnosed with larger tumours were Black [43, 54, 67, 68, 80]. Eight of the studies looking at the relationship between race and tumour size at diagnosis found significant differences in mean and median tumour size at diagnosis, with Black women being diagnosed with significantly larger tumours [8, 38, 43, 74]. Average tumour size for Black women ranged from 1.7 [75] to 3.0 cm [28]. For White women, tumour size ranged from 1.2 [30] to 2.6 cm [38].

Seven studies found no significant difference in tumour size at diagnosis between Black and White women. Method of reporting tumour size was similar to the studies described above. Crowe et al. [57] and Stead et al. [25] found no significant difference in tumour size using the TNM classification system (p > 0.05). Furthermore, Maloney et al. [50] and Swede et al. [62] found no significant difference in mean tumour size at diagnosis between Black and White women (p > 0.05).

### Tumour Grade

Grading of tumours describes the degree of differentiation of tumour cells, with poorly differentiated tumour cells carrying a worse prognosis. In the reviewed literature, tumours were assigned a grade of I (low), II (intermediate), or III (high). Low grade tumours were well differentiated tumours, carrying a more favourable prognosis, while high grade tumours were poorly differentiated. Twenty-eight articles included in this review reported tumour grade/tumour differentiation at the time of diagnosis (Tables 2 and 3). Of these articles, six studies reported no significant difference in tumour grade at the time of diagnosis based on race [8, 22, 30, 39, 74, 78]. Despite the lack of statistical significance, Copson et al. [8] observed that there was a greater proportion of Black women (n = 77/118, 68.1%) diagnosed with grade III tumours compared to White women (n = 1586/2690, 60.4%). Similar findings were reported by Baquet et al. [42], where 43.6% of Black women (n = 6922/15,877) were diagnosed with poorly differentiated breast cancer compared to 29.7% of White women (n = 46,182/155,495). However, the authors did not indicate whether this finding was statistically significant.

**Table 3:** Summary of tumour grade and lymph node involvement at diagnosis for black and white women

References	Location	Sample size	Study design	Study population	Results		How race data was obtained
					Tumour grade	Lymph nodes	
Ambrosone et al. [36]	United States	Cases: 1119 Black: 559 White: 560 Control: 858 Black: 412 White: 446	Multi-center case–control	Women diagnosed with invasive breast cancer or primary DCIS, aged 20 – 75 years	–	Poorly differentiated tumours: Black women – 51.6% White women – 32% (p < 0.05)	Self-report
Barcenas et al. [5]	United States	n = 1,178 Black: 489 White: 670 Asian/Pacific Islander: 12 Other/Unknown: 7	Retrospective	Women diagnosed with breast cancer	Higher proportion of Black women diagnosed with high grade tumours (58.1% Black vs. 36.8% White) (p < 0.001)	–	Not reported
Bowen et al. [11]	United Kingdom	Black: 102 White: 191	Retrospective	Women diagnosed with invasive breast cancer, age ≥ 16 years	Grade I: Black women – 6% vs. White women – 12% (p = 0.02) Grade 3: Black women – 62% vs. White women – 42% (p = 0.02)	No significant difference in lymph node involvement at time of diagnosis	Self-report
Chagpar et al. [74]	United States, Kentucky	n = 1,903 Black: 469 White: 1,145	Retrospective	Women diagnosed with hormone receptor positive breast cancer	No significant difference in tumour grade at diagnosis	–	From Kentucky Cancer Registry
Chlebowski et al. [58]	United States	n = 156,570 Diagnosed with breast cancer: 3938 Black: 242 White: 3,455 Other: 202 Unknown: 39	Prospective	Post-menopausal women aged 50 – 79 years	Poorly differentiated: Black women – 43% vs. White women – 25% Well differentiated: Black women – 13% vs. White women – 25% (p < 0.001)	–	Self-report
Chu et al. [22]	United States	Black: 252 White: 123	Prospective	Low income Black and White women with Stage 0-III, ER- breast cancer, receiving standardized treatment	No significant difference in tumour grade at diagnosis between Black and White women (p = 0.32)	No significant difference in nodal involvement at diagnosis between Black and White women (p = 0.49)	From database
Copson et al. [8]	United Kingdom	n = 2915 Black: 118 White: 2690 Asian: 87	Prospective	Women diagnosed and receiving treatment for breast cancer, aged ≤ 41 years	Grade III: Black women – 68.1% vs. White women – 60.4% (NS)	Positive node involvement: Black women – 56.1% vs. White women – 50.8% (NS)	Self-report
Crowe et al. [57]	United States	Black: 313 White: 2012	Prospective	Women diagnosed with invasive breast cancer with available 2000 census tract data	–	Positive node involvement: Black women—n = 39 vs. White women—n = 32 (p = 0.014)	Self-report
Cunningham et al. [20]	United State, South Carolina and Ohio	South Carolina Black: 5498 White: 18,420 Ohio Black: 6528 White: 64,713	Retrospective	Women of European or African descent aged greater than 15 years diagnosed with invasive breast cancer	Black women diagnosed with Grade III tumours (52–58% vs 37–39% white women) and black women less likely to be diagnosed with grade I tumours (10–14% vs 21–22% p < 0.001)	–	From medical records
DeSantis et al. [4]	United States	n = 193,969 Black: 24,483 White: 169,486	Retrospective	Black and White women (aged between 20 and 99 years)	Black women diagnosed with less differentiated tumours (OR 2.55, 95% CI 2.44–2.66)	Black women diagnosed with lymph node positive tumours (OR 1.44, 95% CI 1.40–1.48)	From medical records
George et al. [67]	United States	Black: 304 White: 330	Retrospective	Black and White women ≤ 85 years	Poorly differentiated: Black women − 42.4% vs. White women − 28.2% (p = 0.0005)	–	From medical records
Iqbal et al. [66]	United States	Black: 38,751 Non-Hispanic White: 268,675 Hispanic White: 34,928 Chinese: 4937 Japanese: 3751 South Asian: 2191 Other Asian: 14,332 Other: 5,998	Retrospective	Women diagnosed with first invasive breast cancer	Distant: Black women – 1.5% vs. White women – 1.0% (p < 0.001)	Positive node involvement: Black women – 24.1% vs. White women – 18.4% (p < 0.001)	From SEER database
Jiagge et al. [82]	United States, Ghana and Ethiopia	Black: 272 White: 321 Ghanaian: 234 Ethiopian: 94	Retrospective	Women diagnosed with invasive breast cancer	Grade I: African American – 12.3% vs. White women – 24.9% Grade II: African American women – 37.3% vs. White women – 41.3% Grade III: African American women – 50.4% vs. White women – 33.7% (p < 0.0001)	–	From medical records
Lund et al. [39]	United States	Black: 176 Non-Black: 23	Retrospective	Women diagnosed with invasive breast cancer	No significant difference in grade at diagnosis between Black and non-Black women (p = 0.099)	–	Self-report
Lund et al. [80]	United States	Black: 814 White: 967	Retrospective	Women diagnosed with primary invasive breast cancer	–	Positive node involvement: Black women – 39.7% vs. White women – 31.1% (p < 0.001)	From Atlanta SEER registry and Georgia Comprehensive Cancer Registry
Maloney et al. [50]	United States	n = 52 Black: 36 White: 16	Retrospective	Women diagnosed with breast cancer, uninsured and below poverty line	–	No significant difference in lymph node involvement at diagnosis for Black women – 19.4% vs. White women – 43.8% (p = 0.068)	From database
McBride et al. [7]	United States	n = 256,174 Black: 21,861 White: 234,313	Retrospective	Women diagnosed with Stage I – IIIa invasive breast cancer	Incidence of high grade tumours: Black women – 45.7% vs. White women – 31.9% (p < 0.0001)	Greater node involvement for Black women – 4.3 vs. White women – 4.0 (p < 0.0001)	From SEER database
Monzavi-Karbassi et al. [64]	United States, Arkansas	Black: 208 White: 869	Retrospective	Black and White women receiving breast cancer treatment	Grade III: Black women 41.8% vs. White women – 4.8% Grade IV: Black women – 35.0% vs. White women – 2.1% (p = 0.02)	–	From Arkansas tumor registry files
Moran et al. [9]	United States	Black: 207 White: 2,164	Retrospective	Women diagnosed with early stage breast cancer	–	Node 2: Black women – 4% White women – 1% (p = 0.0001)	Self-report
Nassar et al. [30]	United States	Black: 217 White: 141	Retrospective	Women diagnosed with primary ductual carcinoma in situ with focal invasion > 1 mm	No significant difference in tumour grade at diagnosis	–	From SEER database and hospital records
Rizzo et al. [28]	United States	Black: 93 Non-Black: 14	Retrospective	Women diagnosed with stage III breast cancer	High grade: Black – 44.3% Non-Black 14.2% (p = 0.04)	–	From SEER database and patient chart
Roberts et al. [63]	United States, North Carolina	Black: 609 White: 859	Retrospective	Women diagnosed with ER^+^, stage I or II and HER2^−^ breast cancer	Grade III: Black women – 24.9% vs. non-Black women – 15.6% (p < 0.001)	–	Self-report
Roseland et al. [10]	United States	Black: 818 White: 1569	Retrospective	Women diagnosed with Stage I, II or III breast cancer	Poorly differentiated: Black women – 45% vs. White women – 32% (p < 0.0001)	Positive node involvement: Black women – 34% vs. White women – 28% (p = 0.0020)	Not reported
Sachdev et al. [78]	United States, Tennessee	Black: 88 White: 36	Retrospective	Women diagnosed and receiving treatment for triple negative invasive breast cancer	No significant difference in tumour grade at diagnosis (p = 0.99)	–	Medical records
Schootman et al. [27]	United States	Black: 2101 White: 32,387 Other: 1320	Retrospective	Women > 66 years diagnosed with distant metastases from primary breast cancer	–	–	From SEER database
Stark et al. [37]	United States	n = 1263 Black: 441 White: 822	Retrospective	Women diagnosed with primary invasive breast cancer	Grade I: Black women – 19.6% White women – 30.3% Grade III: Black women – 45.2% White women – 29.3% (p < 0.001)	No significant difference in lymph node involvement between Black and White women (p = 0.08)	Self-report
Stark et al. [76]	United States, Ghana	Black: 581 White: 1008 Ghanaian women: 75	Retrospective	Women diagnosed with breast cancer	Grade III: African American women – 44.9% vs. White women – 29.3% (p = 0.007)	–	Self-report
Sturtz et al. [70]	United States	n = 160 Black: 62 White: 98	Retrospective	Black and White women diagnosed with triple negative breast cancer	–	No significant difference in lymph node involvement at diagnosis (p = 0.856)	Self-report
Swede et al. [62]	United States, Connecticut	n = 416 Black: 202 White: 214	Retrospective	Women diagnosed and receiving treatment for breast cancer	Grade III/IV: Black women – 50.3% vs. White women – 42.7% (p = 0.04)	No significant difference in the mean number of positive axillary nodes observed for black women and white women (6.67 vs. 3.35) (p = 0.11)	From patient chart
Thomas et al. [60]	United States	Non-hispanic black: 33,301 Non-hispanic white: 241,236 Non-hispanic Asian/Pacific Islander: 9508 Hispanic: 15,782	Retrospective	Women diagnosed with invasive breast cancer	Poorly differentiated: Black women – 47.9% vs. White women – 29.8% (p < 0.001)	–	From National Cancer database
Trivers et al. [16]	United States	n = 476 Black: 116 White: 360	Retrospective	Women diagnosed with unilateral incident invasive breast cancer, aged 20–54 years	–	No significant difference in lymph node involvement was observed between Black women and White women (p = 0.50)	Self-report
Vicini et al. [75]	United States, Michigan	n = 699 Black: 39 White: 660	Retrospective	Women diagnosed with invasive breast cancer	Grade III: Black women – 52% vs. White women – 29% (p = 0.006)	≥ 4 positive lymph nodes: Black women – 18% vs. White women – 8% (p = 0.055)	Self-report
Yang et al. [23]	United States, Florida	n = 935 Black: 130 White: 777 Asian/Pacific Islander/Native American: 13 Not reported: 15	Retrospective	Women diagnosed with inflammatory breast cancer	Black women diagnosed with high grade tumours (92.4%) vs. White women (78.1%) (p = 0.003)	More white women diagnosed with positive lymph node tumours (p = 0.019)	From cancer registry and hospital records
Yankaskas et al. [54]	United States, North Carolina	n = 1691 Black: 380 White: 1311	Retrospective	Women diagnosed with breast cancer, aged ≥ 25 years	Poorly differentiated tumour: Black women – 61.7% White women – 49.3% (p < 0.001)	–	Self-report

Of the articles reviewed, 21 found a significant difference between Black and White women in tumour grade at the time of diagnosis. These studies found that Black women were more likely to be diagnosed with poorly differentiated (grade III) tumours than White women. After adjusting for age, DeSantis et al. [4] found that the odds of Black women being diagnosed with a poorly differentiated tumour was 2.6 times greater than that of White women (OR 2.6, 95% CI 2.4–2.7). In addition, some studies reported that a smaller proportion of Black women were diagnosed with grade I tumours compared to White women. For example, Stark et al. [37] observed that at time of diagnosis 45.2% of Black women (n = 196/441) were diagnosed with grade III tumours and 19.6% were diagnosed with grade I tumours. This significantly differed from White women (n = 232/822), where 29.3% were diagnosed with grade III tumours and 30.3% with grade I tumours (p < 0.001).

### Lymph Node Involvement

Twenty-one of the reviewed studies analyzed differences between Black and White women in relation to lymph node involvement (Table 3). Sixteen of these studies reported nodal involvement as either positive (i.e. at least one lymph node was involved) or negative (i.e. no lymph node involvement). Three studies reported nodal involvement as the average number of positive lymph nodes for Black and White participants [7, 8, 62].

Eleven of the studies reviewed found no significant difference in lymph node involvement by race. Of those studies that found a significant difference in nodal involvement by race, nine indicated a greater likelihood of positive lymph node involvement amongst Black women [4, 5, 7, 10, 57, 80]. Only one study reported a significantly greater likelihood of positive nodal involvement amongst White women with inflammatory breast cancer relative to Black women [12]. Of note, 46.8% of White women (n = 364/777) and 60.0% of Black women (n = 78/130) did not have lymph node involvement [12].

### Tumour Type

Thirty-five out of the 78 reviewed publications assessed the expression of hormone receptors at the time of diagnosis for Black and White women (Table 4). The majority of studies presented findings on the expression of estrogen receptor (ER) and progesterone receptor (PR) and the expression of human epidermal growth factor receptor 2 (HER2) was discussed to a lesser extent. Ten studies reported that there was no significant difference in the expression of ER, PR and HER2, eight studies provided findings on the positive expression of ER and PR and seventeen studies presented results in relation to the negative expression of ER and PR. Twenty studies discussed the occurrence of triple negative breast cancer for Black and White women.

**Table 4:** Summary of hormone status at diagnosis for black and white women

References	Location	Sample size	Study design	Study population	Results			How race data was obtained
					ER/PR	HER2	Triple negative	
Ambrosone et al. [36]	United States	Cases: 1119 Black: 559 White: 560 Control: 858 Black: 412 White: 446	Multi-center case–control	Women diagnosed with invasive breast cancer or primary DCIS, aged 20–75 years	ER^−^: Black – 34.4% White – 22.2% (p < 0.05) PR^−^: Black – 48.3% White – 33.6% (p < 0.05)	–	–	Self-report
Anderson et al. [43]	United States	n = 440,653 Black: 34,478 White: 381,122	Retrospective	Women diagnosed with invasive breast cancer	ER^−^: significantly higher incidence for Black women, IRR = 1.4, 95% CI 1.4–1.4	–	–	From SEER database
Baquet et al.[42]	United States	n = 171,372 Black: 15,877 White: 155,495	Retrospective	Women diagnosed with breast cancer	Black women significantly more likely to be diagnosed with ER−/PR−/HER2− (p < 0.0001)	–	–	From SEER database
Bauer et al. [48]	United States	n = 51,074 Black: 2587 White: 36,671 Other: 11,816	Retrospective	Women diagnosed with primary invasive breast cancer	–	–	Black women significantly more likely to be diagnosed with triple negative tumours vs. White women (OR 1.77, 95% CI 1.59–1.97) Triple negative: Black women – 24.6% vs. white women – 10.8%	From medical record
Bowen et al. [11]	United Kingdom	n = 293 Black: 102 White: 191	Retrospective	Women diagnosed with invasive breast cancer, age ≥ 16 years	ER^−^: Black – 39% White – 21% OR 2.36 (95% CI 1.06–5.00) (p = 0.03)	–	–	Self-report
Brown et al. [41]	United States	n = 61,309 black: 3272 white: 43,398 Other: 14,639	Retrospective	Women diagnosed with primary invasive breast cancer	–	–	Compared to other breast cancers, Black women were diagnosed with a greater proportion of triple negative tumours (10.7%) (p < 0.001) and double negative tumours (7.2%) (p < 0.05)	Medical record
Chen and Li [68]	United States	n = 102,064 Black: 10,874 White: 72,623 Other: 18,567	Retrospective	Women aged ≥ 20 years	–	–	Black – 22.6% White – 10.7%	From SEER database
Copson et al. [8]	United Kingdom	n = 2956 Black: 106 White: 2690	Prospective	Women diagnosed and receiving treatment for breast cancer, aged ≤ 41 years	–	–	Black – 26.1% White – 18.6% (p = 0.043)	Self-report
Crowe et al. [57]	United States	n = 2325 Black: 313 White: 201	Prospective	Women diagnosed with invasive breast cancer with available 2000 census tract data	ER^+^/PR^+^: Black—n = 67 White—n = 82 (p < 0.001)	–	–	Self-report
Cunningham et al. [20]	United States	n = 95,159 Black: 12,026 White: 83,133	Retrospective	Women of European or African descent aged greater than 15 years diagnosed with invasive breast cancer	ER-: Black – 37–40% White – 22–23% PR-: Black – 47–50% White – 33–35% (p < 0.001)	–	–	From medical records
DeSantis et al. [4]	United States	n = 193,969 black: 24,483 White: 169,486	Retrospective	Black and White women (aged between 20 and 99 years)	Black women more likely to be diagnosed with ER/PR negative tumours (OR 2.11, 95% CI 2.04–2.18)	–	Black women more likely to be diagnosed (OR 2.29, 95% CI 2.22–2.37)	From hospital records
George et al. [67]	United States	n = 634 Black: 304 White: 334	Retrospective	Black and White women ≤ 85 years	–	–	Black – 20.1% White – 9.1% (p < 0.0001)	From patient chart
Hahn et al. [47]	United States	n = 829 Black: 250 White: 579	Retrospective	Women diagnosed with unilateral invasive breast cancer	Black women more likely to have ER-/PR- tumours at diagnosis (data not provided)	–	–	Self-report
Hance et al. [56]	United States	n = 180,224 Black: 14,196 White: 155 820	Retrospective	Women diagnosed with breast cancer	For lower grade tumours (non-T4), a greater age-specific incidence rate of ER- tumours was noted amongst black women compared to white women at all ages	–	–	From SEER database
Iqbal et al. [66]	United States	n = 373,563 Black: 38,751 White: 268,675	Retrospective	Women diagnosed with first invasive breast cancer	–	–	≤ 2.0 cm tumours, triple negative: Black – 17.2% White – 8%	From SEER database
Jiagge et al. [82]	United States	Black: 272 White: 321 Ghanaian patients: 234 Ethiopian patients: 94	Retrospective	Women diagnosed with invasive breast cancer	ER^−^: Black – 37.1% White – 19.8% (p < 0.0001) PR^−^: Black – 41.9% White – 25.8% (p < 0.0001)	Black – 81.2% White – 83.3% (p = 0.5088)	–	From medical records
Kenney et al. [40]	United States	n = 184 Black: 70 White: 98 Other: 16	Retrospective	Women with invasive breast cancer	ER + : Black – 70.8% White – 73.2% PR + : Black – 70.8% White – 73.2% (p > 0.05)	HER2 + : Black – 20.8% White – 34.8% (p > 0.05)	–	Self-report
Kwan et al. [13]	United States	n = 2544 Black: 155 White: 1943 Other: 389	Prospective	Women diagnosed with diagnosed with early stage invasive breast cancer, aged 18 – 70 years	–	–	Black – 28.4% White – 10.5% (p < 0.0001)	Self-report
Lund et al. [39]	United States	n = 190 Black: 167 White: 16 Other: 7	Retrospective	Women diagnosed with invasive breast cancer	No significant difference by race. ER: p = 0.109 PR: p = 0.156	No significant difference by race HER2: 0.765	No sig diff in likelihood of having triple negative tumour by race (OR 3.1, 0.8–11.6)	Self-report
Lund et al. [80]	United States	n = 1,842 Black: 814 White: 967	Retrospective	Women diagnosed with primary invasive breast cancer	ER^−^: Black – 32.8% White – 17.7% (p < 0.001) PR^−^: Black – 42.4% White – 27.4% (p < 0.001)	-	Black – 22.6% White – 10.4% (p < 0.001)	From Atlanta SEER registry and Georgia Comprehensive Cancer Registry
Lund et al. [31]	United States	n = 476 Black: 116 White: 360	Retrospective	Women diagnosed with unilateral incident invasive breast cancer, aged 20 – 54 years	No significant difference in likelihood of ER−/PR− tumours by race (OR: 1.3, 0.6–2.6)		Black – 46.6% White – 21.8% p < 0.001	Self-report
Maloney et al. [50]	United States	n = 52 Black: 36 White: 16	Retrospective	Women diagnosed with breast cancer, uninsured and below poverty line	No significant difference by race. ER: p = 0.59 PR: p = 0.76	No significant difference by race HER2: p = 0.85	–	From database
Marti et al. [38]	United States	n = 215 Black: 29 White: 31 Other: 155	Prospective database, retrospective analysis	Women diagnosed with invasive breast cancer or DCIS, of low socioeconomic status	No significant difference in ER/PR expression by race (p > 0.05)	No significant difference in HER2 expression by race (p = 0.56)	–	From medical records
McBride et al. [7]	United States	n = 256,174 Black: 21,861 White: 234,313	Retrospective	Women diagnosed with Stage I–IIIa invasive breast cancer	ER^−^/PR^−^ Black – 27.2% White – 14.6%	–	–	From SEER Database
Moran et al. [9]	United States	n = 2371 Black: 207 White: 2164	Retrospective	Women diagnosed with early stage breast cancer	ER^−^: Black – 54% White – 36% (p = 0.0001) PR^−^: Black – 58% White – 47% (p = 0.0097)	–	Black women – 21% White women – 8% (p < 0.0001)	Self-report
Morris et al. [44]	United States	n = 199,504 Black: 16,853 White: 162,768	Retrospective	Women diagnosed with breast cancer	ER^+^: Black – 51.9% White – 63.1% (p = 0.0003)	–	Black – 20.8% White – 10.4% (p < 0.0001)	From SEER database and hospital records
O’Brien et al. [79]	United States	n = 1149 Black: 518 White: 631	Retrospective	Women diagnosed with invasive breast cancer	ER-: Black – 51% White – 32% PR-: Black – 55% White – 36%	–	–	Self-report
Parise et al. [29]	United States	n = 54,523	Retrospective	Women diagnosed with primary invasive breast cancer	Black women—less likely to be diagnosed with ER +/PR+ tumours OR: 0.80 (95% CI 0.70 – 0.91)	Black women less likely to be diagnosed with HER2- tumours OR 0.69 (95% CI 0.63–0.76)	Black women significantly more likely to be diagnosed OR 1.88 (95% CI 1.69–2.09)	From medical record
Rizzo et al. [28]	United States	n = 107 Black: 93 Non-black: 14	Retrospective	Women diagnosed with stage III breast cancer	No significant difference in ER status (p = 0.25)	–	–	From SEER database and patient chart
Roseland et al. [10]	United States	n = 2387 Black: 818 White: 1569	Retrospective	Women diagnosed with Stage I, II or III breast cancer	ER^−^/PR^−^: Black – 30% White – 19% (p < 0.0001)	–	–	From several databases
Short et al. [77]	United States	n = 575 Black: 99 White: 476	Retrospective	Women newly diagnosed with breast cancer	ER^+^/PR^+^: Black – 56% White – 75% (p = 0.001)			From patient chart
Schootman et al. [27]	United States	n = 3757 Black: 347 White: 3295 Other: 115	Retrospective	Women > 66 years diagnosed with distant metastases from primary breast cancer	ER-: Black: 18.5% White: 12.6% PR-: Black: 26.3% White: 22.5% (no significance data)	–	–	From SEER database
Stark et al. [37]	United States	n = 1263 Black: 441 White: 822	Retrospective	Women diagnosed with primary invasive breast cancer	ER^−^: Black – 35.7% White – 22.1% PR^−^: Black – 45.2% White – 30.1% ER-/PR-: Black – 35.0% White – 21.3% (p < 0.001)	–	Black women more likely to be diagnosed with triple negative tumours (OR 1.72, 1.17–2.54) (p = 0.006)	Self-report
Stead et al. [25]	United States	n = 415 White: 148 Black: 177 Hispanic: 43 Other: 47	Retrospective	Women diagnosed with invasive breast cancer	ER-/PR-: Black – 30.9% White – 17.6% (p < 0.0001)	–	Black – 30% White – 13% (p = 0.0002)	Self-report
Sturtz et al. [70]	United States	n = 160 Black: 62 White: 98	Retrospective	Black and White women diagnosed with triple negative breast cancer	–	–	Black – 28% White – 12% (p < 0.001)	Self-report
Swede et al. [62]	United States	n = 416 Black: 202 White: 214	Retrospective	Women diagnosed and receiving treatment for breast cancer	–	–	Black – 25.7% White – 16.4% (p < 0.01)	From patient chart
Tao et al. [61]	United States	n = 103,498 Black: 9738 White: 93,760	Retrospective	Women diagnosed with invasive breast cancer	–	–	Black – 20% White – 10% (HR 1.21, 95% CI 1.06 – 1.37)	From medical record
Thomas et al. [60]	United States	n = 299,827 Black: 33,301 White: 241,236 Other: 25,290	Retrospective	Women diagnosed with invasive breast cancer	–	–	Black – 24.2% White – 11.4% (p < 0.001)	From National Cancer Database
Trivers et al. [16]	United States	n = 476 Black: 116 White: 360	Retrospective	Women diagnosed with unilateral incident invasive breast cancer, aged 20–54 years	Black women more likely to be diagnosed with ER-/PR- tumours (OR: 1.90, 1.05–3.46, 95% CI)	–	Black women significantly more likely to be diagnosed than white women (OR 2.98, CI 2.12–4.20)	Self-report
Vicini et al. [75]	United States	n = 699 Black: 39 White: 660	Retrospective	Women diagnosed with invasive breast cancer	ER^+^: Black – 44% White – 82% (p < 0.001) PR^+^: Black – 42% White – 65% (p = 0.004)	–	–	Self-report
Woods et al. [2]	United States	n = 5751 Black: 632 White: 5119	Retrospective	Women diagnosed with breast cancer	ER^+^: Black – 64.3% White – 78.5% (p < 0.01) PR^+^: Black – 52.3% White – 65.5% (p < 0.01)	–	–	From patient, patient chart or treating physician
Yankaskas et al. [54]	United States	n = 1691 Black: 380 White: 1311	Retrospective	Women diagnosed with breast cancer, aged ≥ 25 years	ER^+^: Black – 57.8% White – 74.0% (p < 0.001) PR^+^: Black – 50.8% White – 66.3% (p < 0.001)	–	–	Self-report

#### No Significant Difference

As mentioned above, ten studies found no significant difference in the expression of hormone receptors (ER and PR) and HER2 between Black and White women. Lund et al. [39] observed that the frequency of hormone receptor and HER2 expression did not differ between Black and White women (ER, p = 0.109; PR, p = 0.156; HER2, p = 0.765). Furthermore, Rizzo et al. [28] found that the frequency of triple negative breast cancer was not significantly different (p = 0.540). Findings from Chagpar et al. [74] indicate that there was no significant difference in the ER^+^ and PR^+^ tumours for Black and White women, 97.7% (n = 256/469) vs. 97.6% (n = 903/1415), (p = 0.682) and 86.0% (n = 222/469) vs 86.0% (n = 784/1415) (p = 0.873). Four studies found no significant difference in the frequency of HER2 expression for Black and White women [8, 9, 37, 67].

### Positive Hormone Receptor Expression

With regards to the positive expression of ER or PR, four studies observed that a smaller proportion of Black women than White women presented at the time of diagnosis with ER^+^ or PR^+^ tumours [2, 54, 58, 75]. In Vicini et al. [75], 44% of Black women (n = 16/39) were diagnosed with ER^+^ tumours, whereas 82% of White women (n = 430/660) presented with ER^+^ tumours (p < 0.001). The same study found that 42% (n = 15/39) of Black women and 65% (n = 343/660) of White women were diagnosed with PR^+^ tumours (p = 0.004). Crowe et al. [57] and Short et al. [77] explored the expression of ER^+^/PR^+^ tumours in newly diagnosed women in the US. Both studies found that fewer Black women (n = 190/313; n = 40/99) were diagnosed with ER^+^/PR^+^ tumours than White women (n = 1541/2012; n = 267/476) (67.0% vs 82.0%, p < 0.001; 56.3% vs 75.4%, p = 0.001). Using all three markers of hormone receptor expression, Parise et al. [29] assessed differences between Black and White women using the California Cancer Registry. Findings indicate that Black women had a lower odds of being diagnosed with ER^+^/PR^+^/HER2^+^ (OR 0.80, 95% CI 0.70–0.91) and ER^+^/PR^+^/HER2^−^ (OR 0.69, 95% CI 0.63–0.76) tumours when compared to White women.

### Negative Hormone Receptor Expression

Seventeen of the reviewed articles compared the frequency of the absence of ER and PR expression on breast cancer tumours for Black and White women. Eleven studies found a significant difference in the proportion of Black and White women who presented with either ER^−^, PR^−^, or ER^−^/PR^−^ tumours at the time of diagnosis. Findings indicate that the frequency of ER^−^, PR^−^, or ER^−^/PR^−^ tumours was greater for Black women compared to White women. For example, Stead et al. [25] found that a significantly greater proportion of Black women (n = 52/177, 30%) were diagnosed with hormone receptor negative tumours than White women (n = 19/148, 13%) (p < 0.001). Moreover, Trivers et al. [16] and DeSantis et al. [4] found that Black women had a higher odds than White women of being diagnosed with hormone receptor negative (ER−/PR−) tumours (OR 1.90, 95% CI 1.05–3.46, and OR 2.11, 95% CI 2.04–2.18). Stark et al. [37] further observed differences in ER and PR expression. For ER^−^ tumours, the proportion diagnosed was 35.7% for Black women (n = 157/441) and 22.1% for White women (n = 182/822), for PR^−^ tumours the proportions were 45.2% vs 30.1%, and for ER^−^/PR^−^ tumours the proportions were 35.0% vs 21.3% (p < 0.001). A study reporting only ER^−^ tumour status, found that a greater proportion of Black women were diagnosed with ER^−^ tumours (n = 101/272, 37.1%) than White women (n = 63/321, 19.8%) (p < 0.0001) [82]. Similarly, Rizzo et al. [28] observed a significant stage specific difference in the frequency of Black women (n = 47/93, 50.5%) with PR^−^ tumours as compared to White women (n = 3/14, 21.5%) (p = 0.04). Finally, Anderson et al. [43] estimated the incident rate of ER^−^ tumours using SEER databases and found a significantly higher incidence rate among Black women compared to White women (IRR = 1.4, 95% CI 1.4–1.4).

### Triple Negative

Twenty of the articles included in this review explored the incidence of triple negative breast tumours, or tumours that are negative for estrogen, progesterone, or amplified HER2 receptors, by race. Seventeen of these articles found a significant difference in the incidence of triple negative tumours amongst women with breast cancer by race, with a significantly higher likelihood of triple negative tumours amongst Black women with breast cancer. For example, in one study conducted by Trivers et al. [16], Black women were significantly more likely to be diagnosed with triple negative tumours than White women (OR 2.41, 95% CI 1.81–3.21). In the UK, Copson et al. [8] similarly found a significantly higher incidence of triple negative breast cancer amongst Black women relative to White women (n = 30/118, 26.1% vs n = 478/2690, 18.6%, p < 0.05).

Interestingly, only three studies found no significant difference in triple negative tumours by race. For example, Bowen et al. [11] found no significant difference in the likelihood of triple negative tumours by race (p = 0.2) amongst women diagnosed with breast cancer in the UK. In a retrospective study by Lund et al. [39] using data obtained from the SEER Atlanta database, a greater proportion of Black women were diagnosed with triple negative tumours in comparison to non-Black women (n = 49/167, 29.3% vs n = 3/23, 13%), though this difference was not statistically significant (p = 0.05). This study was small, including only 23 non-Black women in a sample size of 190 patients. In contrast, a larger retrospective study by Lund et al. [31] using data from the SEER Atlanta database found that Black women in the US were significantly more likely to be diagnosed with triple negative tumours than White women (OR 1.9, 95% CI 1.2–2.9), even after adjusting for the patient’s age and income, as well as the stage and grade of the breast tumour.

### HER2 Expression

As described above, only ten of the studies included in this narrative review analyzed HER2 expression by race. Overall, no significant difference in HER2 expression was found by race in any of the included studies. The POSH Study, a multi-centre prospective study examining the outcomes of breast cancer in younger women in the UK, found no significant difference in HER2 status by race or ethnicity (p = 0.065). The study did find significant differences by race in other measures, including tumour size at presentation, triple negative tumours, and distant relapse free survival [8]. Nonetheless, there was no significant difference noted by race regarding HER2 status.

In another prospective cohort study by Stark et al. [37], no significant difference was noted in HER2 status between Black and White women in the state of Michigan (n = 110/441, 25.0% Black vs n = 187/822, 22.8% White, p = 0.369). In this study, HER2 status was associated with tumour grade and stage for White women, though no significant interaction was noted for Black women.

## Discussion

The aim of this narrative review was to provide an overview of the differences in breast tumour characteristics in the existing US, UK and Canadian literature for Black and White women (although no Canadian literature was found). Statistically significant differences were found in a number of categories described in this review. In those categories in which differences were found, Black women were consistently at greater risk of high-risk cancer features.

Age at diagnosis differed significantly by race, and Black women were over-represented in younger age groups. Younger age at diagnosis has been consistently linked with more aggressive breast cancers, especially when diagnosis is before 40 years of age [83]. As described above, the method of describing age at diagnosis varied between studies. Most studies looked at the median or mean age at diagnosis for comparison. While statistically significant differences were found, many of these studies reported an average age at diagnosis that was above the age of 50 years [13, 62]. There may be limited clinical application of those findings, given that most breast screening programs begin at the age of 50. In comparison, studies comparing incidence of breast cancer in younger age brackets found that Black women were over-represented in breast cancer diagnoses before the age of 50 [9, 20, 32, 60, 75]. Future studies looking at age of diagnosis should consider the clinical application of this data and include analysis on breast cancer diagnosis prior to 50 years of age.

Interestingly, two of the included studies found that Black women were more likely to be older at age of diagnosis [30, 74]. Nassar et al. [30] found that Black women were more likely to be diagnosed with DCIS at a significantly older age (60 years) compared to White women (56 years, p < 0.001). DCIS, or ductal carcinoma in situ, is an early stage of breast cancer and typically found during breast cancer screening with mammography. This is consistent, therefore, with other studies that found that Black women were more likely to be diagnosed with breast cancer at a later stage of disease and less likely to be diagnosed at an earlier stage, as described above. Chagpar et al. [74] also found that Black women were more likely to be diagnosed at a later age (57 years) compared to White women (55 years, p < 0.05), but they also found that Black women were diagnosed with larger tumours (19 mm vs 17 mm, p = 0.009) at the time of diagnosis. In this single centre study, it can be speculated that Black women in this population were diagnosed with later stage disease at an older age and does not necessarily point to earlier disease in White women.

Fewer studies included stage at diagnosis in their analysis. Nonetheless, in sixteen of those studies which included tumour stage at diagnosis in their analysis, a significant difference was noted by race. Black women were significantly less likely to be diagnosed at earlier stages of cancer (Stage I–II) and were significantly more likely to be diagnosed at a later stage (Stage III–IV). Similarly, Black women were also more likely to have larger, poorly differentiated tumours at the time of diagnosis. Several explanations have been proposed in the literature for these differences. Newman [84] highlighted in the review the role of socioeconomic status in diagnosis of later stage breast cancer in Black women in the United States, despite similar uptake of screening mammography. Barriers to accessing healthcare may result in a delay of tissue diagnosis from the time of an abnormal screening test, for example, resulting in a later stage of disease at the time of diagnosis. However, Newman also argues that race cannot be seen as a substitute for socioeconomic status, pointing to differences in prevalence of more aggressive breast cancer subtypes (e.g. triple negative) by race. Continued research is needed to further elucidate the interaction between race, biology and socioeconomic factors to better interpret the differences in stage at diagnosis by race.

In terms of hormone receptor status, Black women were significantly more likely to be diagnosed with triple negative breast cancer relative to White women. They were also found to be more likely to have estrogen and progesterone receptor negative tumours, but no significant differences in HER2 receptor expression was found by race in any of the included studies. Triple negative breast tumours are not responsive to conventional and currently available targeted therapies and are associated with an overall poorer prognosis [25]. There are some who speculate that this may contribute to the differences in disease prognosis and recurrence by race, along with other tumour characteristics, treatment modalities and certain social factors [6, 8, 31]. Given the importance of targeted therapies in breast cancer management, further research into this area is warranted.

Several limitations of the studies included in this review are noted. The etiology of differences in tumour characteristics for Black and White women appears to be multifactorial, and not fully understood at this time [5]. However, several factors are known to be associated with breast cancer prognosis. These include the tumour traits included in this study, cancer screening uptake and availability [15], socioeconomic status [4, 12], and geography [14]. While many of the included studies included these factors in their analysis, there was significant variation between studies regarding which factors were included and how factors were controlled for.

Interestingly, a number of studies that included certain social and demographic factors found that the impact of race on the prevalence of high-risk cancer features persisted. In a large scale retrospective study using data collected from the National Cancer Database, DeSantis et al. [4] found that Black women were significantly more likely to be diagnosed with metastatic breast cancer relative to White women even after controlling for the independent effects of health insurance status and educational attainment (OR 1.54, 95% CI 1.45–1.63). Similarly, Woods et al. [2] found that Black participants were significantly less likely to have stage I cancer (OR 0.80, 95% CI 0.67–0.96, p = 0.02) at the time of diagnosis, and were significantly more likely to have stage III cancer (OR 1.50, 95% CI 1.11–2.01, p = 0.01) compared to White women, even after controlling for family history, health insurance, smoking, marital status, and whether the participant had reached menopause. Whilst no publications specific to differences in breast cancer prognosis for Black and White women in Canada were identified in this review, a recent Canadian study by Lofters et al. [85] found that immigrant women from Latin America and the Caribbean had a later stage of breast cancer at the time of diagnosis compared to non-immigrants, despite similar access to primary care in two Canadian provinces. It was speculated that there may be a component of genetic susceptibility to aggressive breast cancers amongst women of West African ancestry, given similar findings in studies of African American women in the US [85]. It is, however, challenging to tease these features apart, especially given the lack of race-based data collected in Canada or provided in this study.

In other studies included in this review, the effect of race diminished or was eliminated once social and demographic factors were accounted for. In a retrospective study using data from the SEER Detroit and Los Angeles databases by Lantz et al. [52], Black women were initially found to be significantly less likely to be diagnosed at an earlier stage of breast cancer (Stage I) relative to White women. However, after controlling for age, study site, education, income, and method of detection, no significant difference was found by race (OR 0.79, 95% CI 0.57–1.10). In another smaller single centre retrospective study, no significant difference in age of diagnosis, tumour size, lymph node involvement, or hormone receptor status was found once SES was controlled for [50]. While the current narrative review focused on the incidence of high-risk tumour features by race, it has also highlighted the importance of accounting for social and demographic factors when assessing the impact of these high-risk tumour features on the disparities observed in breast cancer prognosis by race.

Finally, the majority of the studies included in this review did not describe how race information was obtained from participants. Amongst those studies that did describe how this information was obtained, there was significant variability. Methods included self-report [53, 54] and inference based on the race/ethnicity of the participant’s parents, birthplace, surname, or maiden name [41]. The importance of method of reporting race was highlighted in a retrospective study by Boehmer et al. [86], where they compared self-reported race to administrative race data in the context of a dental procedure. They found that administrative data was more likely to be incorrect for individuals who belong to a racial or ethnic group other than White. Future studies investigating breast cancer outcomes by race should make note of the method of reporting of race, as well as the number of individuals for whom race/ethnicity data is missing.

Overall, the literature currently demonstrates significant differences in the prevalence of high-risk breast cancer features by race in the US, and in a few more recent studies conducted in the UK. Given the unique social and political histories of each of these countries, generalizability of these findings to the Canadian context is somewhat limited. Furthermore, as Black is an umbrella term that includes a great deal of diversity, the composition of Black communities differs in each of these countries. Likewise, the impact of health insurance and differing modalities of healthcare delivery on breast cancer outcomes cannot be underestimated. Nonetheless, the findings of this review reinforce the importance of collecting race data in order to identify the impact of structural racism on health outcomes and better inform health screening practices, management guidelines, and to detect and reduce inequity in healthcare outcomes within the Canadian healthcare system.
